# Supplementary figures and images for: Bacterial diversity on larval and female Mansonia spp. from different localities of Porto Velho, Rondonia, Brazil
Source: PLoS One. 2023 Nov 27;18(11):e0293946. doi: 10.1371/journal.pone.0293946 (PMC10681206; doi:10.1371/journal.pone.0293946)

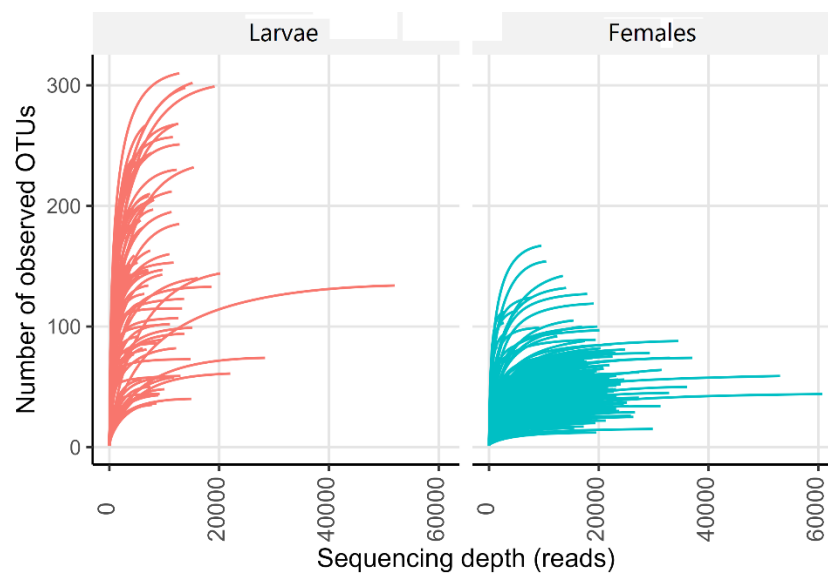

**S3 Fig. Rarefaction curve. ASVs count per given sequencing depth in each sample.**

Supplement: S3 Fig — ASVs count per given sequencing depth in each sample. (PDF) [file pone.0293946.s003.pdf]

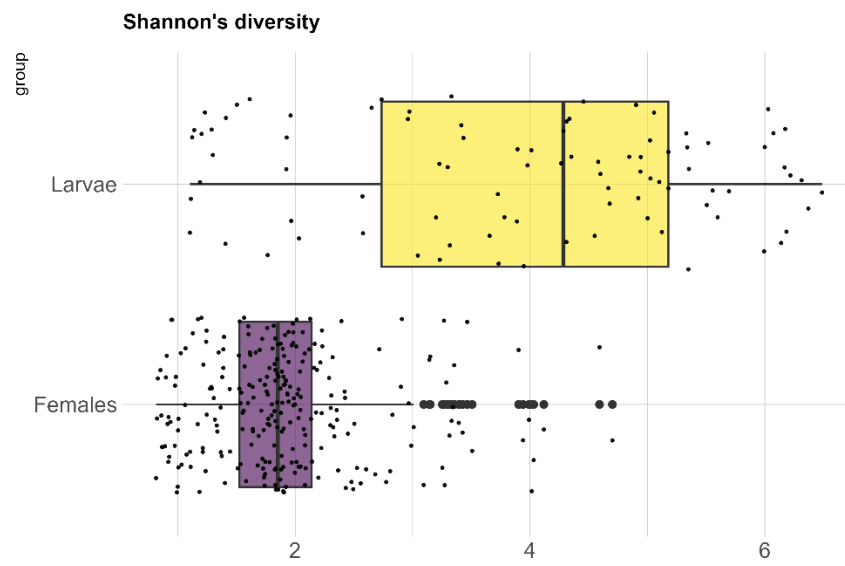

**S4 Fig. Box plot of Shannon indexes of each group (larvae and females).**

Supplement: S4 Fig — (PDF) [file pone.0293946.s004.pdf]
