# Supplementary material for: Bacterial diversity on larval and female Mansonia spp. from different localities of Porto Velho, Rondonia, Brazil
Source: PLoS One. 2023 Nov 27;18(11):e0293946. doi: 10.1371/journal.pone.0293946 (PMC10681206; doi:10.1371/journal.pone.0293946)
Supplement: S5 Fig — (A) PCoA using unweighted Unifrac distance. (B) PCoA using weighted Unifrac distance. (PDF) [file pone.0293946.s005.pdf]

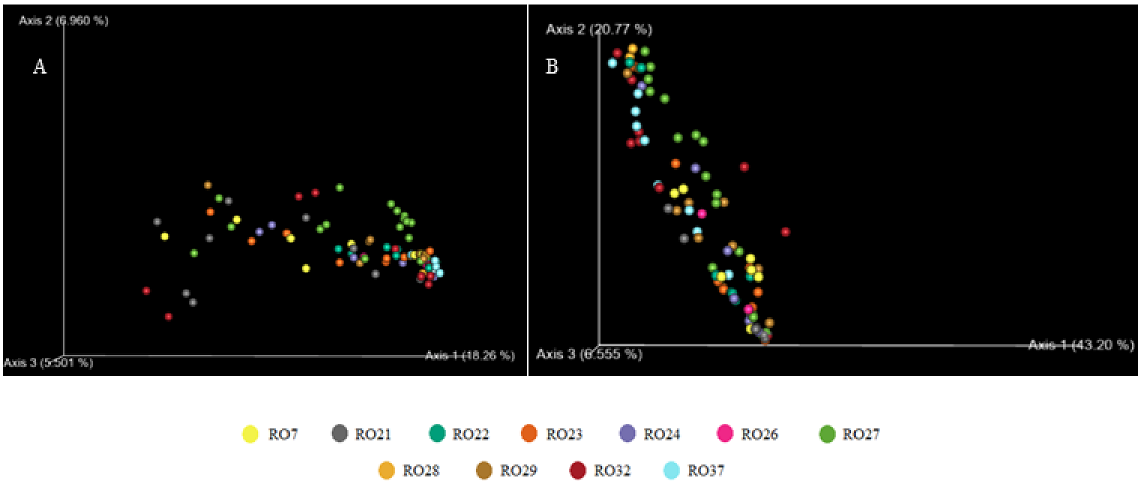

S5 Fig. Principal Coordinate Analysis (PCoA) of the bacterial diversity differences between different larvae collection sites. (A) PCoA using unweighted Unifrac distance. (B) PCoA using weighted Unifrac distance.
