# Supplementary material for: Bacterial diversity on larval and female Mansonia spp. from different localities of Porto Velho, Rondonia, Brazil
Source: PLoS One. 2023 Nov 27;18(11):e0293946. doi: 10.1371/journal.pone.0293946 (PMC10681206; doi:10.1371/journal.pone.0293946)
Supplement: S6 Fig — The color gradient (yellow to purple) represents relative abundance. Yellow: higher bacterial abundance. Purple: lowest bacterial abundance. (PDF) [file pone.0293946.s006.pdf]

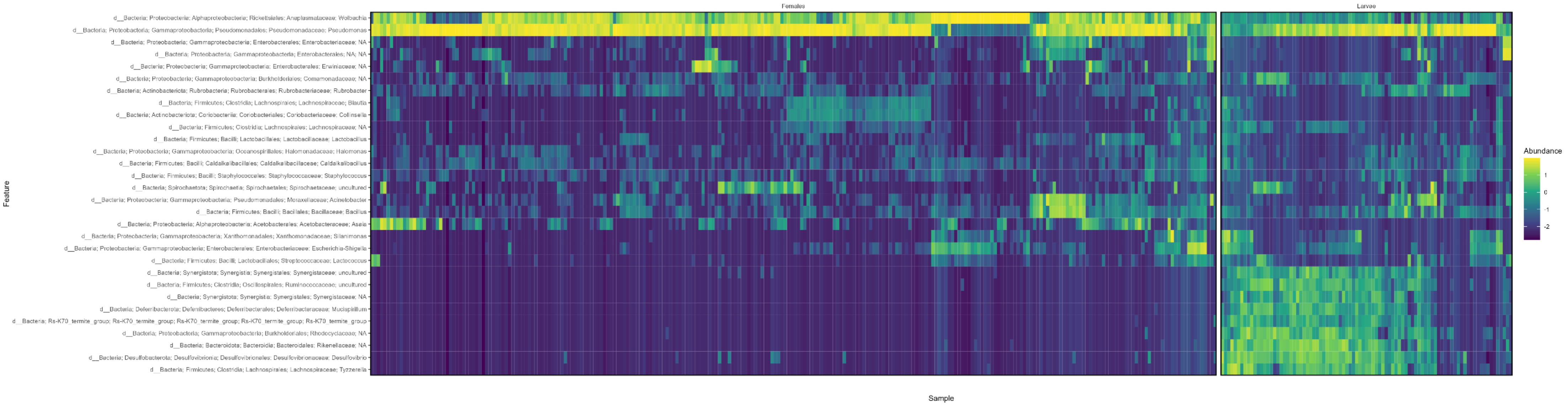

S6 Fig. Heatmap of sequences with taxonomic assignment to genus level. The color gradient (yellow to purple) represents relative abundance.

Yellow: higher bacterial abundance. Purple: lowest bacterial abundance.
