# Supplementary material for: Bacterial diversity on larval and female Mansonia spp. from different localities of Porto Velho, Rondonia, Brazil
Source: PLoS One. 2023 Nov 27;18(11):e0293946. doi: 10.1371/journal.pone.0293946 (PMC10681206; doi:10.1371/journal.pone.0293946)
Supplement: S5 Table — (DOCX) [file pone.0293946.s011.docx]

**S5 Table. Shannon index of each sample.**

| SampleID | shannon_entropy |
| --- | --- |
| 100M | 2.22887291362776 |
| 101M | 0.861586856537745 |
| 102M | 2.42531810269615 |
| 103M | 1.8823225347432 |
| 104M | 2.33395858277103 |
| 105M | 1.9063573202355 |
| 106M | 2.01080198247616 |
| 107M | 1.69970464761992 |
| 108M | 1.00659638408109 |
| 109M | 1.78285192795318 |
| 110M | 1.84914172162456 |
| 111M | 1.44629201028991 |
| 112M | 1.82343561815655 |
| 113M | 2.35427745917661 |
| 114M | 1.84593710876977 |
| 115M | 1.90504062460032 |
| 116M | 3.35469837402827 |
| 117M | 1.92212493876433 |
| 118M | 2.06402270950137 |
| 119M | 1.90266967052506 |
| 120M | 1.87459977418971 |
| 121M | 2.23159413371908 |
| 122M | 1.89121916522179 |
| 124M | 1.80271443708516 |
| 125M | 2.60503316064396 |
| 126M | 2.43169179128303 |
| 127M | 2.22292231846872 |
| 128M | 2.3668827962539 |
| 129M | 2.495052303706 |
| 130M | 1.97085836143233 |
| 131M | 2.14407114669357 |
| 132M | 2.11900746297337 |
| 133M | 1.29205052582309 |
| 134M | 1.91489392960941 |
| 135M | 1.51136053072898 |
| 136M | 1.98577404503679 |
| 137M | 1.98448557185862 |
| 138M | 2.16763587278814 |
| 139M | 2.45004413027311 |
| 140M | 1.9443105065106 |
| 141M | 2.01199388153767 |
| 142M | 1.76092643903985 |
| 143M | 1.82264487850024 |
| 144M | 2.08970254039949 |
| 145M | 2.31945580229887 |
| 146M | 2.4802906245721 |
| 147M | 2.02963029104851 |
| 148M | 1.61057469254905 |
| 149M | 1.87452117890167 |
| 150M | 1.81761134748208 |
| 151M | 1.68119127427372 |
| 152M | 1.92234289683333 |
| 153M | 2.02006599292915 |
| 154M | 1.56976657079164 |
| 155M | 1.36762152216584 |
| 156M | 1.52495331751027 |
| 157M | 1.38898860872248 |
| 158M | 1.20267475772209 |
| 159M | 1.28566591549077 |
| 15L | 3.20045785747663 |
| 160M | 1.24271214138898 |
| 161M | 1.36613818247821 |
| 162M | 1.88858721081237 |
| 163M | 1.11037422167213 |
| 167M | 2.90092187873984 |
| 168M | 1.88723496170112 |
| 169M | 1.93602375386339 |
| 16L | 6.13864515776577 |
| 170M | 2.01356890647902 |
| 171M | 2.12878192943706 |
| 173M | 1.98210056606721 |
| 174M | 2.35681986240012 |
| 175M | 1.92285098868759 |
| 176M | 1.88573090179127 |
| 177M | 1.74688025061084 |
| 178M | 2.04259198152541 |
| 179M | 2.18581105652318 |
| 17L | 4.94042178098106 |
| 180M | 1.76244455720888 |
| 181M | 2.32487331416163 |
| 182M | 1.80808465817665 |
| 183M | 1.63888896649994 |
| 184M | 1.73449108359033 |
| 185M | 1.83066685468982 |
| 186M | 1.5533350600403 |
| 187M | 2.3943922046426 |
| 188M | 1.3170795049807 |
| 189M | 1.68972929355132 |
| 18L | 6.31293420046552 |
| 190M | 2.3002541624612 |
| 191M | 1.52892233385643 |
| 192M | 0.824340056088061 |
| 193M | 2.0446604104867 |
| 194M | 1.53600341642894 |
| 195M | 1.3378943129897 |
| 196M | 3.42616041368309 |
| 197M | 1.52149852048319 |
| 198M | 1.64183157939322 |
| 199M | 2.02990722024965 |
| 19L | 5.99927488965757 |
| 200M | 2.08138445311053 |
| 201M | 3.1505850519266 |
| 202M | 1.37949528621222 |
| 203M | 1.06694821491052 |
| 204M | 0.867042914207598 |
| 205M | 0.928210782185954 |
| 206M | 2.07376894191663 |
| 207M | 0.816150227545133 |
| 208M | 0.892436249188874 |
| 209M | 0.977465383385359 |
| 210M | 0.83066548994623 |
| 211M | 0.975605495718311 |
| 212M | 0.853538694715078 |
| 213M | 0.899996606653551 |
| 214M | 2.68231377205187 |
| 215M | 1.76821212491398 |
| 216M | 0.946215267656733 |
| 217M | 1.66449690779481 |
| 218M | 1.56179542576619 |
| 219M | 1.73255505430907 |
| 220M | 1.64537898090468 |
| 221M | 2.28914548392387 |
| 222M | 1.61466271770762 |
| 223M | 1.83474127289107 |
| 224M | 1.95816702048456 |
| 225M | 1.44342427018868 |
| 226M | 1.30810753220174 |
| 227M | 1.37572080086532 |
| 228M | 1.64047892849153 |
| 229M | 1.34870918348376 |
| 230M | 1.2758344973311 |
| 231M | 1.583139264166 |
| 232M | 1.59853660124604 |
| 233M | 1.78350512018796 |
| 234M | 1.74266282492181 |
| 235M | 2.21480963510057 |
| 236M | 1.75787607930975 |
| 237M | 1.85644315529777 |
| 238M | 1.7015318047388 |
| 239M | 1.60107547553599 |
| 240M | 2.17791340693452 |
| 241M | 1.88044156306499 |
| 242M | 1.78134881989472 |
| 243M | 2.80926270743826 |
| 244M | 2.96730033611441 |
| 245M | 1.38908877867798 |
| 246M | 2.77575166696081 |
| 247M | 1.83151384711348 |
| 248M | 2.0871607109396 |
| 249M | 2.42198338011055 |
| 250M | 1.82619110376936 |
| 251M | 1.83161718800797 |
| 252M | 1.0338883540785 |
| 253M | 1.53536931544005 |
| 254M | 1.89971237528266 |
| 255M | 2.90934097016517 |
| 256M | 1.83236072546472 |
| 257M | 1.72780674184496 |
| 258M | 1.94418519216557 |
| 259M | 1.98018089400828 |
| 260M | 1.99920220072215 |
| 261M | 1.97050319663107 |
| 262M | 1.84831417639728 |
| 263M | 2.08421475893799 |
| 264M | 1.98433815754472 |
| 265M | 1.87801274155158 |
| 266M | 1.76778609451174 |
| 267M | 1.54098166065792 |
| 269M | 1.89532727224661 |
| 271M | 2.22625592867985 |
| 274M | 1.21960632005227 |
| 277M | 2.50427180303511 |
| 278M | 1.90263240093934 |
| 279M | 1.84753396219684 |
| 280M | 1.74449927821732 |
| 281M | 1.85544709869886 |
| 282M | 1.91253502064643 |
| 283M | 1.83594900765392 |
| 284M | 2.30878542582748 |
| 287M | 1.39473753961045 |
| 289M | 3.50759718454921 |
| 290M | 3.26802062603708 |
| 291M | 3.39360661555676 |
| 292M | 3.28747997409921 |
| 293M | 2.30363780447131 |
| 295M | 3.34291514794713 |
| 297M | 3.33305412884808 |
| 299M | 3.31423454207704 |
| 301M | 2.9889526504977 |
| 302M | 3.90337137780862 |
| 306M | 1.77127179417482 |
| 307M | 1.72194779808952 |
| 309M | 1.17029563614708 |
| 314M | 2.04596539614996 |
| 315M | 3.00996617961965 |
| 316M | 2.06507589180772 |
| 319M | 3.94065133297742 |
| 320M | 3.27431271469184 |
| 85M | 1.60336349161394 |
| 87M | 4.70183295504291 |
| 88M | 2.04912317707397 |
| 89M | 1.71225488260783 |
| 90M | 2.13504682425239 |
| 91M | 1.99817854350828 |
| 92M | 4.59201619550111 |
| 93M | 1.71995277865272 |
| 94M | 1.94496623872396 |
| 95M | 2.52686933993363 |
| 96M | 4.0318427142136 |
| 97M | 1.82715023024824 |
| 98M | 1.6282877191737 |
| 99M | 1.74061884501275 |
| 20L | 6.1734842766918 |
| 21L | 6.48670536547066 |
| 22L | 3.97908819838379 |
| 23L | 6.37031007631075 |
| 24L | 6.21765679775592 |
| 25L | 2.97318119212233 |
| 26L | 3.78235429755562 |
| 28L | 1.96161639541752 |
| 29L | 2.651659774001 |
| 30L | 3.72699057848144 |
| 31L | 1.76652785589677 |
| 32L | 3.04513366518817 |
| 33L | 1.61351324213538 |
| 34L | 3.73306821890768 |
| 35L | 3.89372019095631 |
| 36L | 3.33161581748276 |
| 37L | 3.43361692162421 |
| 38L | 3.23236980179285 |
| 39L | 3.22849774611748 |
| 40L | 3.31805586518357 |
| 41L | 1.40724501236445 |
| 42L | 4.9028889710149 |
| 43L | 3.41562826408242 |
| 54L | 5.51782134920008 |
| 55L | 6.07284736328988 |
| 56L | 2.96103247194935 |
| 57L | 1.96769845215412 |
| 58L | 4.55127878495538 |
| 59L | 4.26607561374849 |
| 60L | 4.45548123776868 |
| 61L | 2.73643176528578 |
| 62L | 1.50419821748108 |
| 63L | 3.65585895767616 |
| 64L | 1.28913307900741 |
| 65L | 2.57349967073233 |
| 66L | 3.94723470009027 |
| 67L | 2.57822582839903 |
| 68L | 5.10059097612333 |
| 69L | 4.58135323321285 |
| 70L | 3.29946366188851 |
| 71L | 5.17822107312983 |
| 72L | 4.92244211282073 |
| 73L | 2.0336251912425 |
| 27L | 1.20366053504806 |
| 294M | 2.55829456587356 |
| 296M | 1.95712630000593 |
| 303M | 1.85891675008026 |
| 304M | 3.09501514525576 |
| 305M | 2.82893745949253 |
| 321M | 1.82972707379679 |
| 322M | 1.79991065749774 |
| 323L | 5.05621959550033 |
| 324L | 5.17940402841505 |
| 325L | 5.99321973717303 |
| 326L | 6.02851226367523 |
| 327L | 4.94360374434307 |
| 328L | 4.6670802571999 |
| 329L | 5.33101952293345 |
| 330L | 5.00217441658248 |
| 331L | 4.59990336128688 |
| 332L | 5.12300858887475 |
| 333L | 4.33600890019276 |
| 334L | 5.02235034372144 |
| 335L | 5.69480306727431 |
| 336L | 4.31322901277504 |
| 337L | 4.35232548252595 |
| 338L | 1.10449335116643 |
| 339L | 1.14075001924014 |
| 340L | 5.33880583589446 |
| 341L | 4.67843495804562 |
| 342L | 5.02585113100228 |
| 343L | 3.88934869777644 |
| 344L | 5.59826796010485 |
| 345L | 6.1840442405623 |
| 346L | 5.34868794502945 |
| 347L | 5.35369684122307 |
| 348L | 4.84536826509805 |
| 349L | 6.16791841596489 |
| 350L | 5.55486653223651 |
| 351L | 5.50569298696764 |
| 352L | 4.31028860295354 |
| 353L | 4.01178092689258 |
| 354L | 4.28467161684043 |
| 355L | 1.23234527984086 |
| 356L | 1.92531329144346 |
| 357L | 1.299341238134 |
| 358L | 1.41167369891426 |
| 359L | 1.9285532898256 |
| 360L | 1.11244875600672 |
| 361L | 1.19040853189534 |
| 362L | 1.12376932220968 |
| 373M | 3.4657741044069 |
| 374M | 1.93326214053419 |
| 375M | 2.16169137132041 |
| 376M | 1.24788947959256 |
| 377M | 1.98934033903975 |
| 378M | 2.0292518148154 |
| 379M | 1.90186660387145 |
| 380M | 1.89814521512148 |
| 381M | 1.17321984677084 |
| 382M | 0.95350182024426 |
| 383M | 1.00065707889485 |
| 384M | 4.01543733216636 |
| 385M | 1.24631244955186 |
| 386M | 1.0267970754809 |
| 387M | 1.12935807037072 |
| 388M | 1.10581919117216 |
| 389M | 2.71709190728202 |
| 390M | 1.5661863472276 |
| 391M | 1.61947756132157 |
| 392M | 3.25372940044775 |
| 393M | 1.40259975933939 |
| 394M | 3.99216335542494 |
| 395M | 4.11819090468789 |
| 396M | 2.08522241134538 |
| 397M | 2.19853113322907 |
| 398M | 3.14276665852266 |
| 399M | 0.993745183608568 |
| 400M | 1.55284578042622 |
| 401M | 1.07637730362953 |
| 402M | 1.33293439367394 |
| 403M | 1.00365095927691 |
| 404M | 0.921039097079745 |
| 405M | 1.21369497313299 |
| 406M | 1.21383209593363 |
| 407M | 1.18586887054814 |
| 408M | 1.23782822432068 |
| 409M | 0.984722889588767 |
| 410M | 0.9139775301088 |
| 411M | 0.938858584933708 |
| 412M | 0.869945323784978 |
| 413M | 1.0636178596637 |
| 414M | 0.922732456365011 |
| 415M | 1.00622803360148 |
| 416M | 0.907220581138386 |
