# Supplementary material for: Bacterial diversity on larval and female Mansonia spp. from different localities of Porto Velho, Rondonia, Brazil
Source: PLoS One. 2023 Nov 27;18(11):e0293946. doi: 10.1371/journal.pone.0293946 (PMC10681206; doi:10.1371/journal.pone.0293946)
Supplement: S6 Table — (DOCX) [file pone.0293946.s012.docx]

**S6 Table. Result of pairwise permanova of the bacterial composition in larvae in relation to collection site.**

| **(a)** |  | **Sample size** | **Permutations** | **pseudo-F** | **p-value** | **q-value** |
| --- | --- | --- | --- | --- | --- | --- |
| Group 1 | Group 2 |  |  |  |  |  |
| RO21 | RO22 | 17 | 999 | 4.709553 | **0.018** | 0.070714 |
|  | RO23 | 18 | 999 | 2.375529 | 0.073 | 0.148703 |
|  | RO24 | 16 | 999 | 2.730497 | 0.060 | 0.129038 |
|  | RO26 | 12 | 999 | 1.859958 | 0.057 | 0.129038 |
|  | RO27 | 27 | 999 | 7.135975 | **0.003** | 0.033000 |
|  | RO28 | 12 | 999 | 26.102332 | **0.014** | 0.064166 |
|  | RO29 | 20 | 999 | 4.843687 | **0.002** | 0.027500 |
|  | RO32 | 20 | 999 | 5.809447 | **0.007** | 0.054999 |
|  | RO37 | 20 | 999 | 22.492858 | **0.001** | 0.027500 |
| RO22 | RO23 | 15 | 999 | 0.825362 | 0.414 | 0.549999 |
|  | RO24 | 13 | 999 | 0.670668 | 0.567 | 0.714999 |
|  | RO26 | 9 | 999 | 0.422026 | 0.884 | 0.953333 |
|  | RO27 | 24 | 999 | 1.910857 | 0.110 | 0.201666 |
|  | RO28 | 9 | 999 | 3.616485 | **0.039** | 0.107250 |
|  | RO29 | 17 | 999 | 0.283001 | 0.948 | 0.983773 |
|  | RO32 | 17 | 999 | 1.564015 | 0.151 | 0.239861 |
|  | RO37 | 17 | 999 | 4.667867 | **0.013** | 0.064166 |
| RO23 | RO24 | 14 | 999 | 0.271911 | 0.967 | 0.984907 |
|  | RO26 | 10 | 999 | 0.344024 | 0.944 | 0.983773 |
|  | RO27 | 25 | 999 | 1.976226 | 0.085 | 0.166964 |
|  | RO28 | 10 | 999 | 4.639626 | **0.025** | 0.089375 |
|  | RO29 | 18 | 999 | 0.697038 | 0.608 | 0.728152 |
|  | RO32 | 18 | 999 | 2.057828 | 0.091 | 0.172586 |
|  | RO37 | 18 | 999 | 6.870838 | **0.004** | 0.036666 |
| RO24 | RO26 | 8 | 999 | 0.233357 | 1.000 | 1.000000 |
|  | RO27 | 23 | 999 | 1.535498 | 0.157 | 0.239861 |
|  | RO28 | 8 | 999 | 4.263964 | **0.033** | 0.100833 |
|  | RO29 | 16 | 999 | 0.750781 | 0.572 | 0.714999 |
|  | RO32 | 16 | 999 | 1.457383 | 0.182 | 0.270540 |
|  | RO37 | 16 | 999 | 5.355778 | **0.008** | 0.054999 |
| RO26 | RO27 | 19 | 999 | 0.950881 | 0.420 | 0.549999 |
|  | RO28 | 4 | 999 | 6.159072 | 0.339 | 0.470250 |
|  | RO29 | 12 | 999 | 0.516885 | 0.829 | 0.911899 |
|  | RO32 | 12 | 999 | 0.593608 | 0.609 | 0.728152 |
|  | RO37 | 12 | 999 | 3.348305 | **0.037** | 0.107105 |
| RO27 | RO28 | 19 | 999 | 3.319883 | **0.012** | 0.064166 |
|  | RO29 | 27 | 999 | 1.671976 | 0.137 | 0.235468 |
|  | RO32 | 27 | 999 | 2.266968 | 0.061 | 0.129038 |
|  | RO37 | 27 | 999 | 5.382721 | **0.002** | 0.027500 |
| RO28 | RO29 | 12 | 999 | 2.732004 | **0.045** | 0.112499 |
|  | RO32 | 12 | 999 | 1.854426 | 0.114 | 0.202258 |
|  | RO37 | 12 | 999 | 3.314324 | 0.055 | 0.129038 |
| RO29 | RO32 | 20 | 999 | 1.375690 | 0.203 | 0.293815 |
|  | RO37 | 20 | 999 | 3.879923 | **0.016** | 0.067692 |
| RO32 | RO37 | 20 | 999 | 1.577626 | 0.152 | 0.239861 |
| RO7 | RO21 | 17 | 999 | 3.111467 | **0.009** | 0.054999 |
|  | RO22 | 14 | 999 | 1.185918 | 0.342 | 0.470250 |
|  | RO23 | 15 | 999 | 0.710923 | 0.647 | 0.741354 |
|  | RO24 | 13 | 999 | 0.648899 | 0.709 | 0.795816 |
|  | RO26 | 9 | 999 | 0.661310 | 0.633 | 0.740744 |
|  | RO27 | 24 | 999 | 2.638616 | **0.029** | 0.093823 |
|  | RO28 | 9 | 999 | 4.592340 | **0.026** | 0.089375 |
|  | RO29 | 17 | 999 | 1.552071 | 0.157 | 0.239861 |
|  | RO32 | 17 | 999 | 2.587634 | **0.042** | 0.110000 |
|  | RO37 | 17 | 999 | 7.984605 | **0.002** | 0.027500 |

| **(b)** |  | **Sample size** | **Permutations** | **pseudo-F** | **p-value** | **q-value** |
| --- | --- | --- | --- | --- | --- | --- |
| Group 1 | Group 2 |  |  |  |  |  |
| RO21 | RO22 | 17 | 999 | 3.268832 | **0.004** | 0.022000 |
|  | RO23 | 18 | 999 | 2.218132 | **0.022** | 0.060500 |
|  | RO24 | 16 | 999 | 2.079866 | **0.044** | 0.100833 |
|  | RO26 | 12 | 999 | 1.329607 | 0.223 | 0.308000 |
|  | RO27 | 27 | 999 | 2.616524 | **0.014** | 0.051333 |
|  | RO28 | 12 | 999 | 2.381708 | **0.011** | 0.050416 |
|  | RO29 | 20 | 999 | 3.460051 | **0.001** | 0.006111 |
|  | RO32 | 20 | 999 | 1.246997 | 0.224 | 0.308000 |
|  | RO37 | 20 | 999 | 7.876558 | **0.001** | 0.006111 |
| RO22 | RO23 | 15 | 999 | 1.358861 | 0.175 | 0.253289 |
|  | RO24 | 13 | 999 | 1.306361 | 0.151 | 0.237285 |
|  | RO26 | 9 | 999 | 0.829799 | 0.64 | 0.690196 |
|  | RO27 | 24 | 999 | 2.294936 | **0.01** | 0.049999 |
|  | RO28 | 9 | 999 | 1.158318 | 0.342 | 0.427500 |
|  | RO29 | 17 | 999 | 1.120184 | 0.269 | 0.360853 |
|  | RO32 | 17 | 999 | 2.045276 | **0.019** | 0.058055 |
|  | RO37 | 17 | 999 | 4.431571 | **0.001** | 0.006111 |
| RO23 | RO24 | 14 | 999 | 0.617805 | 0.913 | 0.913000 |
|  | RO26 | 10 | 999 | 0.728429 | 0.885 | 0.901388 |
|  | RO27 | 25 | 999 | 2.065848 | **0.017** | 0.058055 |
|  | RO28 | 10 | 999 | 1.299891 | 0.158 | 0.241388 |
|  | RO29 | 18 | 999 | 1.292254 | 0.164 | 0.243783 |
|  | RO32 | 18 | 999 | 1.884044 | **0.046** | 0.101200 |
|  | RO37 | 18 | 999 | 4.694650 | **0.001** | 0.006111 |
| RO24 | RO26 | 8 | 999 | 0.682059 | 0.818 | 0.848867 |
|  | RO27 | 23 | 999 | 1.403987 | 0.096 | 0.176000 |
|  | RO28 | 8 | 999 | 1.265762 | 0.149 | 0.237285 |
|  | RO29 | 16 | 999 | 0.978151 | 0.429 | 0.481530 |
|  | RO32 | 16 | 999 | 1.412891 | 0.139 | 0.231666 |
|  | RO37 | 16 | 999 | 3.977887 | **0.001** | 0.006111 |
| RO26 | RO27 | 19 | 999 | 0.953237 | 0.365 | 0.436413 |
|  | RO28 | 4 | 999 | 1.803303 | 0.32 | 0.409302 |
|  | RO29 | 12 | 999 | 0.729266 | 0.785 | 0.830288 |
|  | RO32 | 12 | 999 | 0.835983 | 0.429 | 0.481530 |
|  | RO37 | 12 | 999 | 2.988256 | **0.031** | 0.074130 |
| RO27 | RO28 | 19 | 999 | 1.836024 | 0.069 | 0.135535 |
|  | RO29 | 27 | 999 | 2.003588 | **0.018** | 0.058055 |
|  | RO32 | 27 | 999 | 1.472596 | 0.102 | 0.180967 |
|  | RO37 | 27 | 999 | 5.085710 | **0.001** | 0.006111 |
| RO28 | RO29 | 12 | 999 | 1.105702 | 0.364 | 0.436413 |
|  | RO32 | 12 | 999 | 1.606578 | 0.111 | 0.190781 |
|  | RO37 | 12 | 999 | 3.447310 | **0.013** | 0.051071 |
| RO29 | RO32 | 20 | 999 | 2.006281 | **0.031** | 0.074130 |
|  | RO37 | 20 | 999 | 4.126522 | **0.001** | 0.006111 |
| RO32 | RO37 | 20 | 999 | 4.241158 | **0.001** | 0.006111 |
| RO7 | RO21 | 17 | 999 | 1.969676 | **0.022** | 0.060500 |
|  | RO22 | 14 | 999 | 1.874599 | **0.03** | 0.074130 |
|  | RO23 | 15 | 999 | 1.109221 | 0.293 | 0.383690 |
|  | RO24 | 13 | 999 | 1.007988 | 0.391 | 0.457553 |
|  | RO26 | 9 | 999 | 0.917841 | 0.438 | 0.481800 |
|  | RO27 | 24 | 999 | 2.165035 | **0.013** | 0.051071 |
|  | RO28 | 9 | 999 | 1.738590 | **0.049** | 0.103653 |
|  | RO29 | 17 | 999 | 1.552170 | 0.08 | 0.151724 |
|  | RO32 | 17 | 999 | 1.893002 | 0.055 | 0.112037 |
|  | RO37 | 17 | 999 | 6.180241 | **0.001** | 0.006111 |

(a) Weighted and (b) unweighted Unifrac distances. Bold numbers correspond to p < 0.05
