# Supplementary material for: Bacterial diversity on larval and female Mansonia spp. from different localities of Porto Velho, Rondonia, Brazil
Source: PLoS One. 2023 Nov 27;18(11):e0293946. doi: 10.1371/journal.pone.0293946 (PMC10681206; doi:10.1371/journal.pone.0293946)
Supplement: S7 Table — (DOCX) [file pone.0293946.s013.docx]

**S7 Table. Result of the Dunn test for *Desulfovibrio*, *Enhydrobacter* and *Aminomas* in larvae samples collected at different localities.**

***Desulfovibrio***

| Comparison | Z | P.unadj | P.adj |
| --- | --- | --- | --- |
| RO21 - RO22 | -2.06467244 | 3.895400e-02 | 1.000000e+00 |
| RO21 - RO23 | -0.85439508 | 3.928861e-01 | 1.000000e+00 |
| RO22 - RO23 | 1.18289537 | 2.368506e-01 | 1.000000e+00 |
| RO21 - RO24 | -0.96004913 | 3.370305e-01 | 1.000000e+00 |
| RO22 - RO24 | 0.93774733 | 3.483743e-01 | 1.000000e+00 |
| RO23 - RO24 | -0.16755874 | 8.669304e-01 | 1.000000e+00 |
| RO21 - RO26 | -0.48565719 | 6.272102e-01 | 1.000000e+00 |
| RO22 - RO26 | 0.79983321 | 4.238074e-01 | 1.000000e+00 |
| RO23 - RO26 | 0.03679213 | 9.706507e-01 | 1.000000e+00 |
| RO24 - RO26 | 0.14645352 | 8.835634e-01 | 1.000000e+00 |
| RO21 - RO27 | -2.98325316 | 2.852020e-03 | 1.568611e-01 |
| RO22 - RO27 | -0.38171599 | 7.026720e-01 | 1.000000e+00 |
| RO23 - RO27 | -1.82772554 | 6.759075e-02 | 1.000000e+00 |
| RO24 - RO27 | -1.45967569 | 1.443792e-01 | 1.000000e+00 |
| RO26 - RO27 | -1.08718217 | 2.769563e-01 | 1.000000e+00 |
| RO21 - RO28 | -1.82496953 | 6.800563e-02 | 1.000000e+00 |
| RO22 - RO28 | -0.49406544 | 6.212600e-01 | 1.000000e+00 |
| RO23 - RO28 | -1.27546060 | 2.021463e-01 | 1.000000e+00 |
| RO24 - RO28 | -1.12412973 | 2.609580e-01 | 1.000000e+00 |
| RO26 - RO28 | -1.03742688 | 2.995370e-01 | 1.000000e+00 |
| RO27 - RO28 | -0.30059633 | 7.637223e-01 | 1.000000e+00 |
| RO21 - RO29 | -2.07260534 | 3.820902e-02 | 1.000000e+00 |
| RO22 - RO29 | 0.18381375 | 8.541596e-01 | 1.000000e+00 |
| RO23 - RO29 | -1.09967597 | 2.714733e-01 | 1.000000e+00 |
| RO24 - RO29 | -0.83487975 | 4.037854e-01 | 1.000000e+00 |
| RO26 - RO29 | -0.71096207 | 4.771077e-01 | 1.000000e+00 |
| RO27 - RO29 | 0.65744339 | 5.108959e-01 | 1.000000e+00 |
| RO28 - RO29 | 0.62835028 | 5.297745e-01 | 1.000000e+00 |
| RO21 - RO32 | -2.45417285 | 1.412091e-02 | 7.766500e-01 |
| RO22 - RO32 | -0.16245313 | 8.709490e-01 | 1.000000e+00 |
| RO23 - RO32 | -1.45942127 | 1.444492e-01 | 1.000000e+00 |
| RO24 - RO32 | -1.16532691 | 2.438867e-01 | 1.000000e+00 |
| RO26 - RO32 | -0.93126017 | 3.517190e-01 | 1.000000e+00 |
| RO27 - RO32 | 0.22926084 | 8.186662e-01 | 1.000000e+00 |
| RO28 - RO32 | 0.40805217 | 6.832354e-01 | 1.000000e+00 |
| RO29 - RO32 | -0.38156751 | 7.027822e-01 | 1.000000e+00 |
| RO21 - RO37 | -4.86932175 | 1.119819e-06 | **6.159007e-05** |
| RO22 - RO37 | -2.35416504 | 1.856437e-02 | 1.000000e+00 |
| RO23 - RO37 | -3.73644549 | 1.866399e-04 | **1.026519e-02** |
| RO24 - RO37 | -3.25690721 | 1.126332e-03 | 6.194829e-02 |
| RO26 - RO37 | -2.32564704 | 2.003739e-02 | 1.000000e+00 |
| RO27 - RO37 | -2.48094007 | 1.310364e-02 | 7.207002e-01 |
| RO28 - RO37 | -0.98633470 | 3.239689e-01 | 1.000000e+00 |
| RO29 - RO37 | -2.79671641 | 5.162482e-03 | 2.839365e-01 |
| RO32 - RO37 | -2.41514890 | 1.572878e-02 | 8.650828e-01 |
| RO21 - RO7 | -0.51883819 | 6.038736e-01 | 1.000000e+00 |
| RO22 - RO7 | 1.42518876 | 1.541026e-01 | 1.000000e+00 |
| RO23 - RO7 | 0.28903326 | 7.725559e-01 | 1.000000e+00 |
| RO24 - RO7 | 0.43152974 | 6.660832e-01 | 1.000000e+00 |
| RO26 - RO7 | 0.15029263 | 8.805337e-01 | 1.000000e+00 |
| RO27 - RO7 | 2.07802992 | 3.770660e-02 | 1.000000e+00 |
| RO28 - RO7 | 1.44419128 | 1.486852e-01 | 1.000000e+00 |
| RO29 - RO7 | 1.36202051 | 1.731914e-01 | 1.000000e+00 |
| RO32 - RO7 | 1.70828738 | 8.758303e-02 | 1.000000e+00 |
| RO37 - RO7 | 3.89999930 | 9.619297e-05 | **5.290613e-03** |
| *Enhydrobacter* |  |  |  |
| Comparison | **Z** | **P.unadj** | **P.adj** |
| RO21 - RO22 | -0.8929531 | 0.3718823019 | 1.00000000 |
| RO21 - RO23 | -1.5788932 | 0.1143605626 | 1.00000000 |
| RO22 - RO23 | -0.5968181 | 0.5506288345 | 1.00000000 |
| RO21 - RO24 | -2.9169996 | 0.0035341622 | 0.19437892 |
| RO22 - RO24 | -1.9165681 | 0.0552928275 | 1.00000000 |
| RO23 - RO24 | -1.4024273 | 0.1607876727 | 1.00000000 |
| RO21 - RO26 | 0.0000000 | 1.0000000000 | 1.00000000 |
| RO22 - RO26 | 0.5488414 | 0.5831143152 | 1.00000000 |
| RO23 - RO26 | 0.9473359 | 0.3434676416 | 1.00000000 |
| RO24 - RO26 | 1.8448725 | 0.0650560821 | 1.00000000 |
| RO21 - RO27 | -0.3997094 | 0.6893705620 | 1.00000000 |
| RO22 - RO27 | 0.6251709 | 0.5318588773 | 1.00000000 |
| RO23 - RO27 | 1.3752648 | 0.1690493642 | 1.00000000 |
| RO24 - RO27 | 2.8367154 | 0.0045580215 | 0.25069118 |
| RO26 - RO27 | -0.2130907 | 0.8312562361 | 1.00000000 |
| RO21 - RO28 | 0.0000000 | 1.0000000000 | 1.00000000 |
| RO22 - RO28 | 0.5488414 | 0.5831143152 | 1.00000000 |
| RO23 - RO28 | 0.9473359 | 0.3434676416 | 1.00000000 |
| RO24 - RO28 | 1.8448725 | 0.0650560821 | 1.00000000 |
| RO26 - RO28 | 0.0000000 | 1.0000000000 | 1.00000000 |
| RO27 - RO28 | 0.2130907 | 0.8312562361 | 1.00000000 |
| RO21 - RO29 | 0.0000000 | 1.0000000000 | 1.00000000 |
| RO22 - RO29 | 0.8929531 | 0.3718823019 | 1.00000000 |
| RO23 - RO29 | 1.5788932 | 0.1143605626 | 1.00000000 |
| RO24 - RO29 | 2.9169996 | 0.0035341622 | 0.19437892 |
| RO26 - RO29 | 0.0000000 | 1.0000000000 | 1.00000000 |
| RO27 - RO29 | 0.3997094 | 0.6893705620 | 1.00000000 |
| RO28 - RO29 | 0.0000000 | 1.0000000000 | 1.00000000 |
| RO21 - RO32 | 0.0000000 | 1.0000000000 | 1.00000000 |
| RO22 - RO32 | 0.8929531 | 0.3718823019 | 1.00000000 |
| RO23 - RO32 | 1.5788932 | 0.1143605626 | 1.00000000 |
| RO24 - RO32 | 2.9169996 | 0.0035341622 | 0.19437892 |
| RO26 - RO32 | 0.0000000 | 1.0000000000 | 1.00000000 |
| RO27 - RO32 | 0.3997094 | 0.6893705620 | 1.00000000 |
| RO28 - RO32 | 0.0000000 | 1.0000000000 | 1.00000000 |
| RO29 - RO32 | 0.0000000 | 1.0000000000 | 1.00000000 |
| RO21 - RO37 | 0.0000000 | 1.0000000000 | 1.00000000 |
| RO22 - RO37 | 0.8929531 | 0.3718823019 | 1.00000000 |
| RO23 - RO37 | 1.5788932 | 0.1143605626 | 1.00000000 |
| RO24 - RO37 | 2.9169996 | 0.0035341622 | 0.19437892 |
| RO26 - RO37 | 0.0000000 | 1.0000000000 | 1.00000000 |
| RO27 - RO37 | 0.3997094 | 0.6893705620 | 1.00000000 |
| RO28 - RO37 | 0.0000000 | 1.0000000000 | 1.00000000 |
| RO29 - RO37 | 0.0000000 | 1.0000000000 | 1.00000000 |
| RO32 - RO37 | 0.0000000 | 1.0000000000 | 1.00000000 |
| RO21 - RO7 | -3.5718123 | 0.0003545195 | **0.01949857** |
| RO22 - RO7 | -2.4697862 | 0.0135193835 | 0.74356609 |
| RO23 - RO7 | -1.9539661 | 0.0507052268 | 1.00000000 |
| RO24 - RO7 | -0.4563257 | 0.6481557633 | 1.00000000 |
| RO26 - RO7 | -2.1953655 | 0.0281373906 | 1.00000000 |
|  |  |  |  |
| RO27 - RO7 | -3.5648045 | 0.0003641275 | **0.02002701** |
| RO28 - RO7 | -2.1953655 | 0.0281373906 | 1.00000000 |
| RO29 - RO7 | -3.5718123 | 0.0003545195 | **0.01949857** |
| RO32 - RO7 | -3.5718123 | 0.0003545195 | **0.01949857** |
| RO37 - RO7 | -3.5718123 | 0.0003545195 | **0.01949857** |
| *Aminomonas* |  |  |  |
| Comparison | **Z** | **P.unadj** | **P.adj** |
| RO21 - RO22 | -0.97255303 | 0.330775498 | 1.0000000 |
| RO21 - RO23 | -0.02451416 | 0.980442491 | 1.0000000 |
| RO22 - RO23 | 0.90358811 | 0.366213846 | 1.0000000 |
| RO21 - RO24 | -0.81716376 | 0.413834851 | 1.0000000 |
| RO22 - RO24 | 0.10298904 | 0.917971667 | 1.0000000 |
| RO23 - RO24 | -0.75982697 | 0.447358019 | 1.0000000 |
| RO21 - RO26 | -1.57166959 | 0.116027198 | 1.0000000 |
| RO22 - RO26 | -0.92061069 | 0.357253721 | 1.0000000 |
| RO23 - RO26 | -1.52520692 | 0.127207494 | 1.0000000 |
| RO24 - RO26 | -0.97419695 | 0.329958758 | 1.0000000 |
| RO21 - RO27 | -0.84443648 | 0.398425542 | 1.0000000 |
| RO22 - RO27 | 0.31786343 | 0.750588532 | 1.0000000 |
| RO23 - RO27 | -0.75779610 | 0.448573056 | 1.0000000 |
| RO24 - RO27 | 0.17995075 | 0.857191236 | 1.0000000 |
| RO26 - RO27 | 1.17836323 | 0.238651828 | 1.0000000 |
| RO21 - RO28 | -2.83788180 | 0.004541400 | 0.2497770 |
| RO22 - RO28 | -2.14388790 | 0.032041870 | 1.0000000 |
| RO23 - RO28 | -2.76583645 | 0.005677700 | 0.3122735 |
| RO24 - RO28 | -2.17543133 | 0.029597809 | 1.0000000 |
| RO26 - RO28 | -0.98080376 | 0.326689523 | 1.0000000 |
| RO27 - RO28 | -2.49039630 | 0.012760073 | 0.7018040 |
| RO21 - RO29 | -2.41019730 | 0.015943896 | 0.8769143 |
| RO22 - RO29 | -1.21466538 | 0.224493740 | 1.0000000 |
| RO23 - RO29 | -2.24784165 | 0.024586289 | 1.0000000 |
| RO24 - RO29 | -1.27012833 | 0.204038921 | 1.0000000 |
| RO26 - RO29 | 0.18014153 | 0.857041459 | 1.0000000 |
| RO27 - RO29 | -1.86020791 | 0.062856117 | 1.0000000 |
| RO28 - RO29 | 1.44635374 | 0.148078009 | 1.0000000 |
| RO21 - RO32 | -0.34818985 | 0.727697607 | 1.0000000 |
| RO22 - RO32 | 0.65657588 | 0.511453660 | 1.0000000 |
| RO23 - RO32 | -0.30376239 | 0.761308926 | 1.0000000 |
| RO24 - RO32 | 0.51562250 | 0.606118093 | 1.0000000 |
| RO26 - RO32 | 1.37064209 | 0.170486557 | 1.0000000 |
| RO27 - RO32 | 0.45370925 | 0.650038111 | 1.0000000 |
| RO28 - RO32 | 2.63685430 | 0.008367876 | 0.4602332 |
| RO29 - RO32 | 2.06200745 | 0.039207027 | 1.0000000 |
| RO21 - RO37 | -3.06135755 | 0.002203358 | 0.1211847 |
| RO22 - RO37 | -1.80558368 | 0.070983394 | 1.0000000 |
| RO23 - RO37 | -2.86176142 | 0.004212939 | 0.2317117 |
| RO24 - RO37 | -1.83404965 | 0.066646625 | 1.0000000 |
| RO26 - RO37 | -0.19580601 | 0.844762004 | 1.0000000 |
| RO27 - RO37 | -2.59091859 | 0.009572013 | 0.5264607 |
| RO28 - RO37 | 1.07040620 | 0.284436510 | 1.0000000 |
| RO29 - RO37 | -0.65116025 | 0.514943049 | 1.0000000 |
| RO32 - RO37 | -2.71316769 | 0.006664337 | 0.3665385 |
| RO21 - RO7 | -1.01358902 | 0.310778899 | 1.0000000 |
| RO22 - RO7 | -0.03783332 | 0.969820581 | 1.0000000 |
| RO23 - RO7 | -0.94266220 | 0.345853716 | 1.0000000 |
| RO24 - RO7 | -0.13933812 | 0.889182970 | 1.0000000 |
| RO26 - RO7 | 0.89538848 | 0.370579459 | 1.0000000 |
| RO27 - RO7 | -0.36289409 | 0.716684005 | 1.0000000 |
| RO28 - RO7 | 2.11866569 | 0.034118730 | 1.0000000 |
| RO29 - RO7 | 1.17362939 | 0.240543503 | 1.0000000 |
| RO32 - RO7 | -0.69761188 | 0.485419953 | 1.0000000 |
| RO37 - RO7 | 1.76454769 | 0.077639814 | 1.0000000 |

Values marked in bold correspond to p.adj < 0.05.
