# Supplementary material for: Bacterial diversity on larval and female Mansonia spp. from different localities of Porto Velho, Rondonia, Brazil
Source: PLoS One. 2023 Nov 27;18(11):e0293946. doi: 10.1371/journal.pone.0293946 (PMC10681206; doi:10.1371/journal.pone.0293946)
Supplement: Attachment — Submitted filename: Response to Reviewers.docx [file pone.0293946.s015.docx]

**Reviewer #1:**

**6. The larvae were not identified beyond genus level. However, the adult females were. The authors need to use the extracted DNA to sequence and identify the larvae at species level. Mosquito larvae of different species can have significant differences in their microbiota even if they are reared in the same breeding site. Thus, treating all the larvae as Mansonia spp. and comparing them to adult females of different Mansonia species is not a good variable for the microbiota analyses.**

**Thank you for this comment. We agreed on the importance of identifying the larvae at the species level, however, unfortunately we were unable to carry out this step, as there is no more genetic material from the larval specimens. Analyzes of the bacterial diversity between larvae and adults were carried out considering only the genus, without considering the species of the adults.**

**I strongly encourage the authors to add this point to the manuscript as one of the limitations of the study.**

*Thank you for this observation. We added the following text: "It is largely known that geographic location, aquatic ecosystem biotic and abiotic factors, and mosquito species can influence on the composition of the microbiota [23,25,59]. Thus, this study faces a limitation in understanding the bacterial difference found between the adults and larvae because the later life stages were identified to the genus level. Likely, geographic location and the mosquito species may have contributed to the differences observed in the bacterial composition of the larvae and females." (Lines 327-332).*

**Reviewer #2:**

**Introduction:
Lines 49-51: This text is repetitive - "Mosquitoes contain a range of symbiotic microorganisms that are crucial to their development [10]. The interactions between mosquitoes and bacteria are crucial for larval development [11]."**

*Thank you for this comment. We changed the text to "Mosquitoes contain a range of symbiotic microorganisms that are crucial to their development [10]. The interactions between mosquitoes and bacteria during larval development can stimulate metabolic changes with functional consequences on adult fitness [11]." (Lines 50-53).*

**Lines 55-57: Please rephrase to "These microorganisms can be obtained by the larvae from the environment, primarily from water in aquatic habitats, and vertically [19-21].**

*Thank you for this observation. The sentence was reformulated according to your suggestion: "These microorganisms can be obtained by the larvae from the environment, primarily from water in aquatic habitats, and vertically [19-21]." (Lines 57-59).*

**Lines 67-68: Please rephrase to "Symbiotic bacteria can be used as a potential tool for both mosquito and pathogen control."**

*Thank you for your kind attention. The text was rewritten as suggested: "Symbiotic bacteria can be used as a potential tool for both mosquito and pathogen control [26,27]." (Lines 69-70).*

**Lines 69-70: The phrase "As the composition of microbiota in adult mosquitoes is derived from their larval stages and water of the breeding [21], changes in the larval microbiota impact not only larval but also adult development" is confusing - what do the authors mean by "adult development"?**

*Thank you for your careful attention. We wanted to refer to adult fitness. We changed the text to "As the composition of microbiota in adult mosquitoes is derived from their larval stages and water of the breeding [21], changes in the larval microbiota can impact not only larval development but also adult fitness [10,11]." (Lines 70-72).*

**Lines 79-82: I suggest "verify differences in bacterial composition associated with the larvae and females between areas in the vicinities of Santo Antonio Energia hydroelectric power plant (SAE) reservoir in the municipality of Porto Velho, Rondonia state, Brazil".**

*Thank you for this suggestion. The sentence was reformulated according to your suggestion: "verify differences in bacterial composition associated with the larvae and females between areas in the vicinities of Santo Antonio Energia hydroelectric power plant (SAE) reservoir in the municipality of Porto Velho, Rondonia state, Brazil." (Lines 81-83).*

**Methods:
Line 89: Please clarify if larvae and adult mosquitoes were collected in the same period**.

*Thank you for this observation. We added the following sentence: "Collections were conducted approximately every four months from October 2018 to March 2020 (S2 Table)." (Lines 89-91).*

**Results:
Please indicate female species in Table 1 (could be inside parentheses as it is shown for plant species).**

*Thank you for this suggestion. We added the names of the analyzed species at each ID locality in Table 1 and changed the footnote to: "Value inside parentheses in “Host plant species" column shows the number of larvae analyzed from each host plant species in each locality." (Lines 188-189).*

**Discussion:
The collection of larvae and adults in different places might have contributed to differences seen in the microbiota between different life stages. This must be considered in the discussion.**

*Thank you for this observation. We added the following text: "It is largely known that geographic location, aquatic ecosystem biotic and abiotic factors, and mosquito species can influence on the composition of the microbiota [23,25,59]. Thus, this study faces a limitation in understanding the bacterial difference found between the adults and larvae because the later life stages were identified to the genus level. Likely, geographic location and the mosquito species may have contributed to the differences observed in the bacterial composition of the larvae and females." (Lines 327-332).*

**Lines 253-255: Please rephrase to "Although Mansonia mosquitoes are not considered the primary vectors of arboviruses, some were found naturally infected with the Western Equine Encephalitis virus in Argentina".**

*Thank you for this comment. We followed your suggestion and now it reads: "Although Mansonia mosquitoes are not considered the primary vectors of arboviruses, some were found naturally infected with the Western Equine Encephalitis virus in Argentina [39]." (Lines 255-257).*

**Line 262-265: Please rephrase to "bacterial communities observed during the larvae stage of Mansonia species may vary from those of culicids with distinct ecology".**

*Thank you for this observation. The text was changed as suggested: "the bacterial communities observed during the larvae stage of Mansonia species may vary from those of culicids with distinct ecology." (Lines 264-266).*

**Line 266-267: What do the authors mean by "a connected bacterial grouping"?**

*Thank you for this observation. This sentence was not clear and we changed the text to: "The bacterial genus Desulfovibrio contains gram-negative sulfate-reducing bacteria, which are present in organic-rich environments and are linked to water bio-corrosion [47]. This bacterial genus has been identified in the larvae of Mansonia spp. studied and other insects [48,49]." (Lines 266-269).*

**Lines 268-269: Please rephrase to "The frequency of Desulfovibrio in larvae (3.59%) was higher than in adults (0.005%)."**

*Thank you for this comment. The sentence was rewritten and now reads is: "The frequency of Desulfovibrio in larvae (3.59%) was higher than in adults (0.005%)." (Lines 269-270).*

**Line 269: The 16S sequencing allow the comparison of bacteria relative abundance between groups of samples, so it is not possible to say that "Mansonia spp. the larvae hosted a larger quantity of the sulfur-reducing bacteria of the genus Sulfurospirillum" (please modify to "larger relative amount" or similar). The same is true for "… were found in large numbers" (line 271).**

*Thank you for this observation. We changed the text to: "Besides Desulfovibrio, Mansonia spp. larvae hosted a larger relative amount of the sulfur-reducing bacteria of the genus Sulfurospirillum [50]. The Aminomonas bacteria from the Synergistaceae family were found in large relative amount in the larvae, however, no contigs were recognized in Mansonia females." (Lines 270-274).*

**Line 276: Please rephrase to "The vegetation root microhabitat fosters anaerobic bacteria, such as Aminomonas".**

*Thank you for this observation. We changed the sentence as suggested: "The vegetation root microhabitat fosters anaerobic bacteria, such as Aminomonas." (Lines 277-278).*

**Line 333: What do the authors mean by "insect populations"? Maybe you mean "insect species".**

*Thank you for this comment. Exact, like "insect species". We changed to: "Estimated that approximately 65% of the insect species are infected with Wolbachia [65]." (Line 342).*
